# Machine Learning-Based Prediction Framework for Complex Neuromorphic Dynamics of Third-Order Memristive Neurons at the Edge of Chaos

**DOI:** 10.3390/e28010042

**Published:** 2025-12-29

**Authors:** Tao Luo, Lin Yan, Weiqing Liu

**Affiliations:** 1 School of Science, Jiangxi University of Science and Technology, Ganzhou 341000, China; 6720231234@mail.jxust.edu.cn (T.L.); 6720231225@mail.jxust.edu.cn (L.Y.); 2 Key Laboratory of Low Dimensional Quantum Materials and Sensor Devices of Jiangxi Education Institutes, Ganzhou 341000, China

**Keywords:** memristor, third-order memristive neurons, edge of chaos dynamics, next-generation reservoir computing, neuromorphic pattern prediction, partial state observability

## Abstract

As conventional computing architectures face fundamental physical limitations and the von Neumann bottleneck constrains computational efficiency, neuromorphic systems have emerged as a promising paradigm for next-generation information processing. Memristive neurons, particularly third-order circuits operating near the edge of chaos, exhibit rich neuromorphic dynamics that closely mimic biological neural activities but present significant prediction challenges due to their complex nonlinear behavior. Current approaches typically require complete system state measurements, which is often impractical in real-world neuromorphic hardware implementations where only partial state information is accessible. This paper addresses this critical limitation by proposing an innovative hybrid machine learning framework that integrates a Modified Next-Generation Reservoir Computing (MNGRC) with XGBoost regression. The core novelty lies in its dual-path prediction architecture designed specifically for partial state observability scenarios. The primary path employs NGRC to capture and forecast the system’s temporal dynamics using available state variables and input stimuli, while the secondary path leverages XGBoost as an efficient state estimator to infer unobserved state variables from minimal measurements. This strategic combination enables accurate prediction of diverse neuromorphic patterns with significantly reduced sensor requirements. Experimentally, the framework demonstrates its capability to identify and predict the complex spectrum of neuromorphic behaviors exhibited by the third-order memristive neuron. This includes accurately capturing all 18 distinct neuronal patterns, which are theoretically grounded in Hopf bifurcation analysis near the edge of chaos. Additionally, the framework successfully addresses the inverse problem of input stimulus reconstruction. By achieving accurate prediction of complex dynamics from limited states, our approach represents a key breakthrough, where full state access is often impossible, thereby addressing a critical challenge in edge AI and brain-inspired computing.

## 1. Introduction

As traditional transistor-based computing architectures approach fundamental physical limits and the von Neumann bottleneck increasingly constrains computational efficiency, neuromorphic computing has emerged as a promising paradigm for next-generation reservoir computing (NGRC) [1]. In contrast to conventional systems that separate memory and processing, neuromorphic architectures integrate storage with computation while operating at substantially lower power. This makes them well-suited for applications in edge AI and brain-inspired computing. Specifically, NGRC leverages neuromorphic hardware for efficient temporal signal processing and dynamic pattern recognition at the edge, as well as for simulating neural dynamics and synaptic plasticity in brain-inspired computational models [2,3].

Memristive neurons have emerged as a promising hardware platform for neuromorphic computing, leveraging their intrinsic nonlinear dynamics to authentically emulate biological neural processes [3]. Recent advances in memristor-coupled neural systems have demonstrated remarkable capabilities in generating complex dynamical behaviors. For example, Yu et al. [4] achieved synchronization of multi-scroll attractors in non-polynomial memristive Hopfield neural networks with efficient FPGA implementation, while He et al. [5] demonstrated hidden chaotic dual-wing attractors in fractional-order memristive Hopfield networks. With the aid of sophisticated nonlinear analysis methods to characterize system dynamics [6]. Further innovations include biologically inspired architectures, such as the work by Stasenko et al. [7], who demonstrated astrocyte-mediated control of bursting modes in spiking neuron networks with memristor-implemented plasticity, reflecting fundamental principles of synaptic memory storage [8]. Liang et al. [9] revealed how memristor non-volatility influences synchronization in heterogeneous coupled neurons, and Lai and Qin [10] developed extremely simple cyclic memristive chaotic neural networks with effective synchronization control. At the architectural frontier, Lin et al. [11] developed triple-memristor Hopfield neural networks exhibiting space-dependent multi-structure attractors with initial-offset sensitivity, and Zhenlong et al. [12] identified spiking and bursting discharge behaviors in memristor-based oscillators. These principles have been extended to practical applications: Feng et al. [13] integrated fractional-order Hopfield neural networks with differentiated encryption for medical image protection, and Qian et al. [14] developed robust memristor-enhanced polynomial hyper-chaotic maps for multi-channel image encryption. Most recently, state-dependent variable fractional-order hyperchaotic dynamics in coupled systems have enabled novel approaches to high-performance image protection [15,16,17].

Third-order memristive neuron circuits, in particular, exhibit a rich repertoire of neuro-morphic behaviors—from periodic spiking and bursting oscillations to chaotic dynamics—especially near the edge of chaos (EoC) [16]. This complexity emerges from the delicate stability in locally active memristors (LAMs), where minute parameter shifts can trigger significant dynamical transitions via Hopf bifurcations [14]. Recent studies demonstrate these circuits can generate up to 18 distinct neuromorphic states under varied stimuli and configurations [15,16], spanning resting states, sub-threshold oscillations, adaptive spiking, and biologically plausible bursting patterns. These phenomena are rigorously characterized through Chua’s local activity theory and zero-pole trajectory analysis of neuronal admittance functions [16,17,18], with experimental validation across diverse memristive materials and circuit topologies [17,18,19,20].

Despite these advances, accurately predicting the dynamical responses of memristive neurons to arbitrary inputs remains challenging. Traditional simulation approaches, which solve nonlinear differential equations, become computationally intensive for long-term dynamics or input optimization tasks. Machine learning techniques, particularly reservoir computing and gradient boosting methods, offer compelling alternatives for modeling such complex systems [21]. These approaches have demonstrated high effectiveness in chaos prediction, time-series forecasting, and neural dynamics modeling while maintaining computational efficiency [22].

Critically, while recent breakthroughs in memristive chaotic networks [8,9,10,11,12,13,14,15,16,17] have pushed hardware implementation boundaries, they predominantly focus on forward dynamics analysis rather than inverse control—leaving a gap in predicting or prescribing inputs for target neuromorphic responses under partial observability. This study bridges neuromorphic hardware with machine learning by introducing a predictive framework specifically designed for third-order memristive neurons operating near the EoC. In contrast to conventional approaches that require complete system state information, our hybrid model integrates next-generation reservoir computing (NGRC) with XGBoost regression to predict output dynamics using minimal state information [21,22,23,24,25]. This strategy not only achieves high predictive accuracy but also addresses the inverse problem—determining the required input patterns to produce specific neuromorphic responses—an essential capability for practical neuromorphic applications where full state observability is often impractical [26,27,28,29,30].

The primary contributions of this work are as follows:

- A novel hybrid machine learning framework integrating improved Next-Generation. Reservoir Computing (MNGRC) with XGBoost regression is proposed for predicting complex neuromorphic dynamics of third-order memristive neurons operating near the edge of chaos. The core innovation lies in the dual-path prediction architecture specifically designed for partial state observability scenarios.

- By training exclusively on a single periodic spiking behavior, our MNGRC architecture successfully captures the fundamental dynamics necessary to predict transitions between qualitatively different states, including the shift from periodic spiking to chaotic oscillations and the critical transition across supercritical Hopf bifurcation boundaries between self-sustained oscillations and stable resting states.
- The framework represents an advance by predicting 18 neuromorphic behaviors based on partial state variables, overcoming the previous limitation which depended on complete system state measurements.
- The framework effectively solves the inverse problem by determining required input stimuli from observed neuronal responses.

## 2. Materials and Methods

To address the critical challenge of predicting the multifaceted dynamics of memristive neuromorphic systems, this section introduces a novel Dual-stride hybrid forecasting framework. The central premise of our approach is the hierarchical integration of two next-generation reservoir computing (NGRC) units, each engineered to distill dynamical features from distinct temporal scales. This architecture (MNGRC) is specifically designed to capture the wide spectrum of neuromorphic behaviors, from fast spiking to slow bursting oscillations, which are intrinsically Dual-stride in nature.

The XGBoost module is pivotal for its exceptional capability in cross-state prediction. It is rigorously demonstrated that the XGBoost regressor can accurately infer the classifications and full-state dynamics of the system by observing only a single state variable (e.g., membrane potential) when trained on the rich feature space created by the MNGRC This ability to reconstruct the complete system behavior from partial observations is a key innovation.

### 2.1. Next-Generation Reservoir Computer

This module systematically constructs delayed embeddings of the input signal and applies nonlinear transformations to explicitly reconstruct the system’s short-term dynamics [31,32]. For a d-dimensional input vector $u(t)\in R^{d}$ at time t, the i-th variable $u_{i}={\left[u_{1,i},u_{2,i},\cdots ,u_{d,i}\right]}^{T}$ is first mapped into a linear delay embedding.

(1) $$O_{lin}=u_{i}⊕u_{i-s}⊕u_{i-2s}⊕\cdots ⊕u_{i-(k-1)s}\;,$$

where $O_{lin}$ denotes the linear feature component generated via discrete sampling, where each term corresponds to the state at current and previous steps spaced by interval s. Subsequently, nonlinear features $O_{nonlin}$ are formed by concatenating these linear vectors across all time steps *i*.

(2) $$O_{nonlin}=\overset{P}{\overset{⏠}{O_{lin}⊗O_{lin}⊗O_{lin}⊕\cdots ⊗O_{lin}}}\;,$$

where ⊗ denotes the outer product between vectors. The number of linear feature vectors subjected to outer product operations is determined by the chosen polynomial order *P*, which also governs the maximum degree of monomials generated in each operation [33,34,35].

Finally, the final nonlinear polynomial feature vector $O_{\mathrm{all}}$$O_{\mathrm{all}}$ is constructed by concatenating the nonlinear features $O_{nonlin}$, linear features $O_{lin}$, and a bias term R.

(3) $$O_{\mathrm{all}}=O_{nonlin}⊕O_{lin}⊕R\;,$$

**Figure 1:**
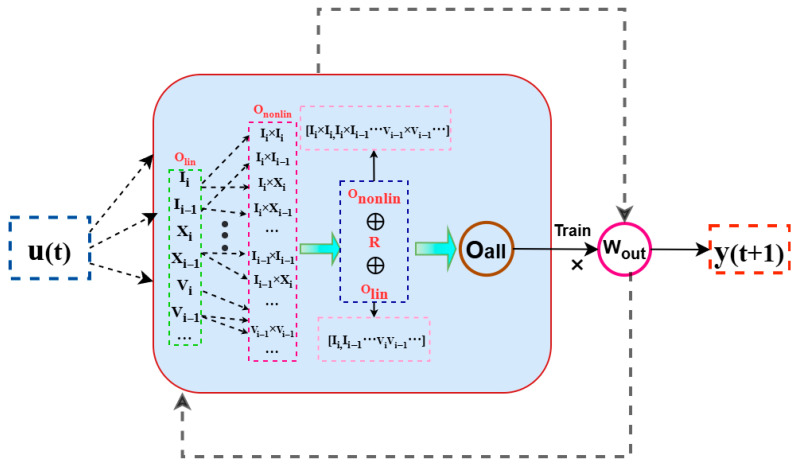
Structure diagram of next-generation reservoir computer.

In both the training and prediction phases, the NGRC framework adheres to a computational procedure analogous to conventional Reservoir Computing (RC). The core of the process involves a linear readout as shown in Figure 1.

During the training phase, the output matrix $W_{out}$ is obtained by applying ridge regression to the collected high-dimensional feature vectors (or states), with the target being the corresponding system outputs. This ridge regression step is crucial for ensuring numerical stability and preventing over fitting.

During the prediction phase, the output is generated by a straightforward linear transformation: the newly computed feature vector is multiplied by the trained $W_{out}$ matrix. This process is computationally efficient, since it avoids the need for further iterative optimization during inference [36,37,38].

### 2.2. eXtreme Gradient Boosting

XGBoost (eXtreme Gradient Boosting) is an optimized implementation of the gradient boosting algorithm that efficiently handles large-scale data and delivers high predictive accuracy. The core principle involves iteratively building an ensemble of decision trees where each subsequent tree corrects the errors of the previous ones.

**Figure 2:**
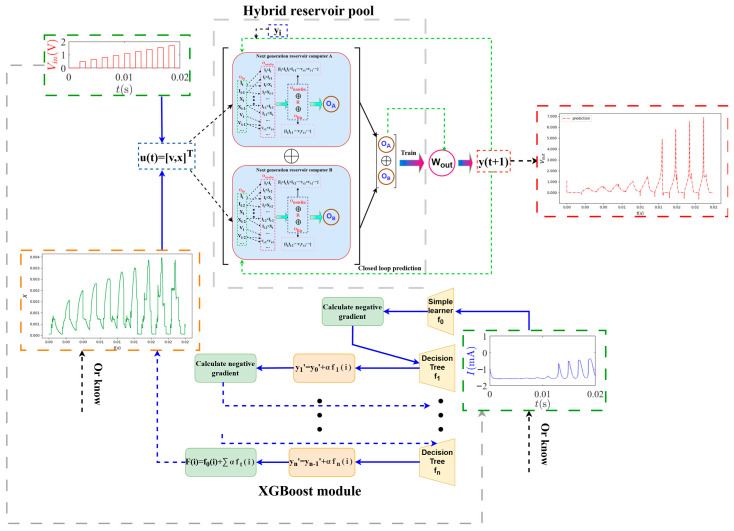
Hybrid machine learning framework for predicting complex neuromorphic dynamics of third-order memristive neurons. The architecture integrates a dual-path prediction system: the XGBoost module (lower part) estimates unknown state variable *x* using input voltage $V_{in}$ while the hybrid reservoir pool (upper part) generates high-dimensional features from the joint input vector $u(t)={\left[V_{in},x\right]}^{T}$. Three distinct input patterns are processed with reservoir reset before each signal type to ensure clean state evolution. The trained readout weights $W_{out}$ generate the output prediction $V_{out}$ by multiplying with the feature vector from closed-loop reservoir operation.

The fundamental working mechanism of XGBoost can be understood through its iterative process as shown in Figure 2. XGBoost begins with a simple learner $f_{0}$ that provides an initial prediction for all samples during training. For each iteration t=1,2,…,T, the algorithm consists of the following stages: (i) Calculates the negative gradients of the loss function with respect to the current predictions. (ii) Fits a decision tree $f_{t}$ to these negative gradients. (iii) Updates the model predictions by adding the contribution from the new tree, weighted by a learning rate parameter α.

The model prediction at iteration t is defined as:

(4) $${\hat{y}}_{i}=\sum_{m=1}^{M}f_{m}(x_{i})={{\hat{y}}_{i}}^{\left(t-1\right)}+\alpha f_{t}(x_{i})\;,$$

where $f_{t}(x_{i})$ represents the *t*-th decision tree, and α is the learning rate that controls the contribution of each tree to prevent overfitting, ${\hat{y}}_{i}$ is the predicted value of the *i*th sample $x_{i}$.

The objective function of XGBoost consists of loss function $l(y_{i},{\hat{y}}_{i})$ and regularization term $\Omega (f_{k})$.

(5) $$\zeta (\theta )=\sum_{i}l(y_{i},{\hat{y}}_{i})+\sum_{k}\Omega (f_{k})=\sum_{i=1}^{n}l(y_{i},{{\hat{y}}_{i}}^{\left(t-1\right)}+\alpha f_{t}(x_{i}))+\sum_{k}\Omega (f_{k})\;,$$

(6) $$\Omega (f_{k})=\gamma T+\frac{1}{2}\lambda \sum_{j=1}^{T}{\omega_{j}}^{2}\;,$$

where ζ(θ) is the expression of linear space, T is the number of leaves in tree *k*, $\omega_{j}$ represents the score on leaf j, γ and λ are regularization parameters.

A key innovation of XGBoost is the use of second-order Taylor expansion to approximate the loss function, which provides more accurate optimization compared to first-order methods used in traditional Gradient Boosting Decision Tree (GBDT).

(7) $$\zeta^{\left(t\right)}\approx \sum_{i=1}^{n}\left[l(y_{i},{{\hat{y}}_{i}}^{\left(t-1\right)}+\alpha g_{i}f_{t}(x_{i})+\frac{1}{2}h_{i}f_{t}{\left(x_{i}\right)}^{2}\right)]+\Omega (f_{t})\;,$$

(8) $$g_{i}=\frac{\partial l(y_{i},{{\hat{y}}_{i}}^{\left(t-1\right)})}{\partial ({{\hat{y}}_{i}}^{\left(t-1\right)})}\;,$$

(9) $$h_{i}=\frac{\partial^{2}l(y_{i},{{\hat{y}}_{i}}^{\left(t-1\right)})}{\partial {\left({{\hat{y}}_{i}}^{\left(t-1\right)}\right)}^{2}}\;,$$

where $g_{i}$ is the first-order gradient and $h_{i}$ is the second-order gradient. By differentiating the approximated objective function with respect to $f_{t}(x)$ and setting the derivative to zero, the optimal prediction value for leaf j can be derived.

(10) $$w_{j}^{*}=-\frac{\sum_{i\in I_{j}}g_{i}}{\sum_{i\in I_{j}}h_{i}+\lambda }\;,$$

(11) $$Obj=-\frac{1}{2}\sum_{j=1}^{T}\frac{{g_{i}}^{2}}{h_{j}+\lambda }+\gamma T\;,$$

where $I_{j}$ represents the set of instances assigned to leaf j. $w_{j}^{*}$ is the optimal weight of each leaf node. Obj is the optimal target value.

### 2.3. Machine Learning Prediction Framework

The proposed hybrid machine learning framework represents a significant advancement in predicting the complex neuromorphic dynamics of third-order memristive neurons operating near the edge of chaos. The core innovation lies not merely in adding a parameter channel, but in the comprehensive prediction architecture that integrates next-generation reservoir computing (NGRC) with extreme gradient boosting (XGBoost) to address the fundamental challenge of state variable unobservability in memristive neuronal systems.

The framework’s unique contribution is its dual-path prediction architecture with a hybrid reservoir pool, which strategically combines two NGRC modules with distinct topological structures but identical parameter settings (Different spacing intervals, denoted as S). This hybrid approach preserves critical temporal features while capturing diverse aspects of the neuromorphic dynamics. The first NGRC module processes input signals with standard interval spacing, while the second employs a different spacing pattern, allowing the system to characterize both short-term and long-term temporal dependencies in the neuronal dynamics.

A key feature of the framework is the parameter channel that integrates both input voltage $V_{in}$ and internal state variable x or i (x is used here.) into a joint input vector $u(t)={\left[V_{in},x\right]}^{T}$.

The input signals, namely the current state u(t) and its two delayed versions u(t−m) and u(t−n) (with *m* = 1 and *n* = 2 as defined in this work), are fed into two parallel Next-Generation Reservoir Computing (NGRC) units, denoted as NGRC A and NGRC B, respectively. Each reservoir nonlinearly maps its respective input stream into a high-dimensional feature space, producing distinct feature vectors $O_{A}$ and $O_{B}$. Subsequently, these two feature vectors are concatenated to form a comprehensive, fused feature representation $O_{fin}$, which integrates Dual-stride temporal information for downstream processing.

(12) $$O_{fin}=O_{A}⊕O_{B}={\left[O_{A},O_{B}\right]}^{T}\;,$$

Finally, the predicted output y(t+1) is obtained by linearly combining the reservoir state $O_{fin}$ with the readout weights $W_{out}$, learned via minimization of a loss function:

(13) $$W_{out}=U^{\prime }\cdot O^{T}\cdot {\left(O\cdot O^{T}+\gamma \cdot I\right)}^{-1}\;,$$

(14) $$y(t+1)=W_{out}\cdot O_{fin}\;,$$

where $U^{\prime }$ and O denote the matrix forms of u(t) and $O_{fin}$, respectively; γ>0 is the L2 regularization coefficient; and I is the identity matrix.

The framework employs three distinct input voltage signal configurations for comprehensive training. Crucially, before generating feature vectors for each input type, the reservoir is reset and reinitialized to ensure clean state evolution [39,40,41,42,43]. This reset process eliminates cross-contamination between different signal types and maintains the integrity of the temporal dynamics representation for each specific signal configuration.

When the internal state x is unknown, the XGBoost module serves as a predictive estimator to approximate this critical variable. The XGBoost algorithm computes optimal leaf weights $w_{j}^{*}$ using the Formula (10). The predicted state variable $\hat{x}$ is then fed back into the reservoir pool along with $V_{in}$ to generate the final prediction of the output voltage $V_{out}$. This two-stage prediction pipeline significantly improves prediction accuracy compared to direct prediction methods, especially for complex behaviors such as chaotic spiking and periodic bursting.

The detailed architecture of the proposed machine learning prediction framework is depicted in Figure 2.

## 3. Results

### 3.1. Prediction of Chaotic Dynamic Behavior of Third Order Memristor Neuron

**Figure 3:**
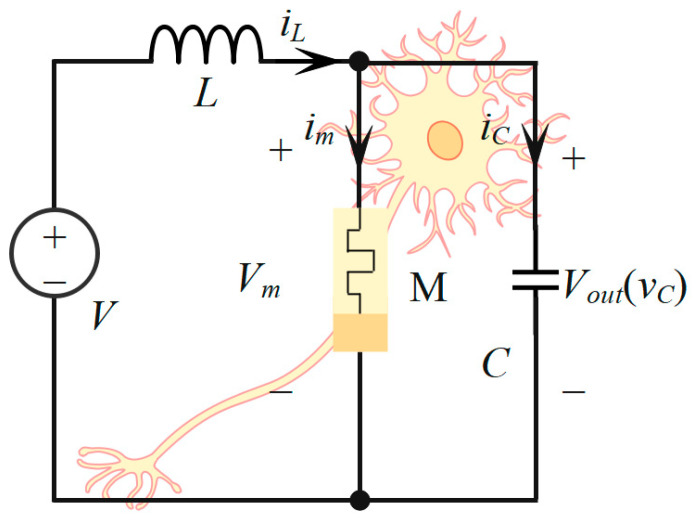
The circuit topology of third-order memristive neuron circuit.

A third-order neuron circuit was constructed by Lili Huang et al. [16], as shown in Figure 3. They presented a novel third-order memristive neuron circuit operating at the edge of chaos (EoC), designed using a locally active memristor (LAM) model based on Chua’s local activity theory. This third-order system demonstrates rich neuromorphic dynamics by operating near the edge of chaos (EoC), with its behavior governed by the interplay between the locally active memristor and passive components.

We first conduct a comprehensive evaluation of the proposed machine learning architecture’s performance using this third-order system, thereby validating its efficacy and practical utility for modeling complex nonlinear dynamics.

The complete third-order memristive neuron circuit is mathematically described by the following state equations:

(15) $$\left\{\begin{array}{l} \dot{x}=-2x-10+v_{c}\\ \dot{i}=\frac{1}{L}(V-v_{c})\\ {\dot{v}}_{c}=\frac{1}{C}(i_{L}-x^{2}v_{c}) \end{array}\right.\;,$$

where the system’s state is characterized by x, the capacitance voltage $v_{c}$, the inductance current i, and the external excitation V. The measured output $V_{out}$ is taken directly across the capacitor, thus $V_{out}=v_{c}$.

The numerical simulations of the third-order memristive neuron system were conducted using the 4th order Runge–Kutta (RK4) integration method with a fixed time step of dt=0.01, corresponding to a total simulation duration of 200 s (20,000 data points). To eliminate transient effects, the initial 10,000 data points (100 s) were discarded, and the remaining 10,000 points were used for analysis. To enhance model robustness, Gaussian noise with a mean of 0 and a standard deviation of $1\times 10^{-6}$ is added to the data. The system dynamics were characterized by varying the excitation voltage (V) across the critical range where complex neuromorphic behaviors emerge. Specifically, three representative voltage values (*V* = 7.202 V, 7.3 V, and 7.8 V) were selected to demonstrate periodic-2 spiking, periodic-4 spiking, and chaotic oscillation, respectively. All state variables were scaled to the [0, 1] range using min-max normalization, ensuring consistent feature distribution for both training and testing data. This normalization procedure was applied uniformly to all simulation results to facilitate comparative analysis while preserving the intrinsic dynamical properties. The circuit parameters were fixed at L=0.08H and C=0.04F, with initial conditions set to [x(0),i(0),v(0)]=[0,0,0].

During the training phase, the model was trained with time series obtained under an excitation voltage of V = 7.202 V, which exhibits periodic-2 spiking dynamics. For each prediction task under different *V*, we implemented a systematic protocol to ensure forecasting reliability. The reservoir states were systematically reset before each prediction trial to eliminate potential state contamination from previous computations. This reset procedure was complemented by a dedicated preheating phase, where approximately 20% of the initial transient response was discarded to allow the reservoir system to reach stable operational conditions. This preheating strategy effectively addressed the challenge of initial condition sensitivity commonly encountered in chaotic and complex nonlinear systems [36,37,38,39,40,41,42,43,44,45,46].

The prediction framework employed a hybrid stacked architecture (MNGRC) comprising two distinct next-generation reservoir computing units—NGRC A and NGRC B—operating at different temporal resolutions. This Dual-stride configuration was specifically designed to capture both fast spiking dynamics and slower bursting patterns characteristic of memristive neurons.

**Figure 4:**
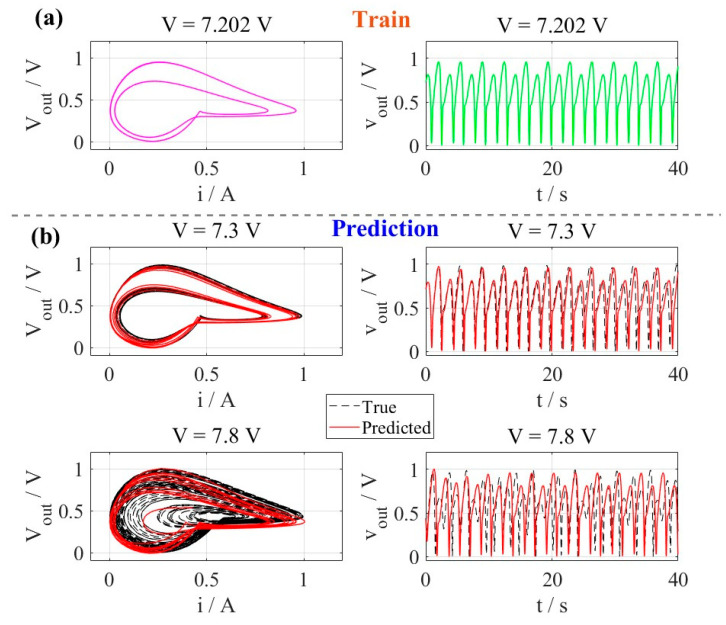
MNGRC prediction of memristive neuron dynamics: (**a**) Training on periodic-2 spiking at *V* = 7.202 V; (**b**) Generalization to periodic-4 spiking (*V* = 7.3 V) and chaotic oscillation (*V* = 7.8 V), with predicted trajectories closely matching ground truth.

When evaluated on two previously unseen voltage conditions—*V* = 7.3 V (periodic-4 spiking) and *V* = 7.8 V (chaotic oscillation)—the trained model demonstrated exceptional generalization capability. The phase portrait reveals that the predicted trajectory accurately captures the characteristic periodic-4 spiking pattern, while the time-domain analysis confirms precise reproduction of both oscillation frequency and amplitude for *V* = 7.3 V. More significantly, the model maintained high prediction accuracy in the challenging chaotic regime at *V* = 7.8 V, where the phase space exhibits complex attractor structures with inherent sensitivity to initial conditions, as shown in Figure 4. The close correspondence between predicted (solid red) and actual (dashed black) trajectories indicates that the MNGRC framework successfully learned the fundamental dynamical principles governing the memristive neuron’s behavior across different operational regimes.

Building upon these qualitative findings, we further quantified the system’s dynamical properties by computing the maximum Lyapunov exponent λ. Under the periodic-4 spiking regime (V = 7.3 V), the predicted time series yielded a small positive $\lambda_{pre}\approx 0.022$, and the true maximum Lyapunov exponent $\lambda_{true}\approx 0.041$. Crucially, in the chaotic regime (V = 7.8 V), the model’s prediction produced a larger positive $\lambda_{pre}\approx 0.1$, and the true maximum Lyapunov exponent $\lambda_{true}\approx 0.09$, reflecting the expected increase in exponential divergence. This rise in the predicted Lyapunov exponent—from the periodic to the chaotic regime confirms that the MNGRC framework captures the transition to deterministic chaos, as a positive $\lambda_{pre}$ inherently signifies chaotic dynamics.

The operating points at *V* = 8.6 V and *V* = 8.8 V represent distinct dynamical regimes near the supercritical Hopf bifurcation boundary, providing an excellent framework for studying the transition between oscillatory and stable states. At *V* = 8.6 V, the memristive neuron circuit exhibits self-sustained oscillations with a characteristic frequency of approximately 4.83 rad/s, However, the system converges to a stable resting state at *V* = 8.8 V. This strategic selection allows for comprehensive analysis of the system’s behavior across the critical transition boundary. The initial conditions for the third-order neuron system are the same as above. Similarly, 10,000 data points are numerically obtained from the neuron system stimulated with input voltages *V* = 8.59 V (oscillating state) and *V* = 8.79 V (stable resting state) by the RK4 solver (with time step being dt = 0.01) after 3000 transient states to train the model, respectively. During the prediction phase, the network was reset to its initial condition, and a segment of the transient response was utilized to guide the prediction process, enabling accurate characterization of the system’s dynamical properties near the edge of chaos.

**Figure 5:**
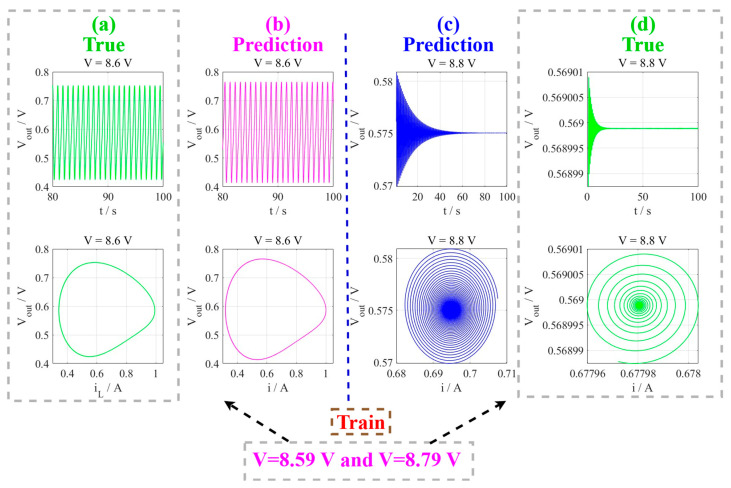
MNGRC prediction of third-order memristive neuron dynamics: (**a**–**d**) Cross-regime generalization from training at *V* = 8.59 V and *V* = 8.79 V to (**a**,**b**) periodic oscillations at V = 8.6 V and (**c**,**d**) stable point behavior at V = 8.8 V, with predicted trajectories closely matching dynamical behavior in both time-domain and phase space.

The MNGRC framework demonstrates exceptional generalization capabilities in predicting the complex dynamics of the third-order memristive neuron system. As illustrated in Figure 5, the model trained on two reference voltages (*V* = 8.59 V and *V* = 8.79 V) successfully captures the distinct behaviors at *V* = 8.6 V (self-sustained oscillations) and *V* = 8.8 V (stable resting point) without additional training. The prediction exhibits remarkable fidelity to the original system dynamics, with RMSE values of 0.276 and 0.171, respectively. The predicted trajectories closely follow the true dynamics, preserving critical features such as oscillation frequency and amplitude for the periodic behavior at V = 8.6 V, and accurately converging to the equilibrium point for the stable state at *V* = 8.8 V. A comparison of the stable equilibrium points shows close correspondence between the MGRC model (0.23, 0.69, 0.57) and the actual system (0.24, 0.67, 0.56). This observed discrepancy in steady-state prediction highlights an area for potential improvement in the current modeling approach [46,47,48,49,50,51].

The hyperparameter configuration for the MNGRC framework is systematically presented in Table 1, where NGRCA and NGRCB represent the two reservoir computing modules with distinct topological structures but identical parameter settings. The selection of *p* = 4 was specifically chosen to adequately represent the intricate nonlinear relationships that emerge across the full spectrum of neuromorphic behaviors, from resting states to chaotic spiking. Unlike arbitrary parameter selection, this value was systematically determined through Bayesian optimization [52,53,54,55,56]. Through rigorous Bayesian optimization, the polynomial order parameter P for both reservoirs, providing optimal balance between representational capacity and computational efficiency across all tested dynamical regimes. This unified parameter selection was validated through extensive cross-validation across the entire operational range of the third-order memristive neuron system (3.5–10 V), demonstrating consistent performance in capturing both simple periodic oscillations and complex chaotic behaviors near the edge of chaos.

Other parameters, including the input delay k=2, stride values $s_{A}=1$ and $s_{B}=1$, and ridge regularization $\gamma =3.4\times 10^{-4}$, were maintained at fixed values throughout all experiments. This consistent configuration highlights the robustness of these settings for predicting diverse memristive neuron dynamics, as the framework successfully generalizes across all 18 distinct neuromorphic behaviors identified in the system. The fixed parameter approach ensures methodological consistency while the adaptive selection of the polynomial order demonstrates the framework’s flexibility in matching model complexity to the underlying dynamics of the system.

### 3.2. Prediction of 18 Forms of Patterns

The MNGRC framework exhibits exceptional generalization capabilities across diverse operational regimes of the third-order memristive neuron system. By training on data from a single voltage condition, the model successfully captures the fundamental dynamics without requiring retraining for new input conditions. The dual-path architecture leverages a hybrid reservoir pool to extract high-dimensional temporal features while the XGBoost module estimates critical state variables from partial information.

For each prediction task, a systematic protocol is implemented: reservoir states are reset before each trial to eliminate state contamination, followed by a preheating phase that discards approximately 20% of the initial transient response. This approach effectively addresses the sensitivity to initial conditions characteristic of nonlinear systems operating near the edge of chaos.

**Figure 6:**
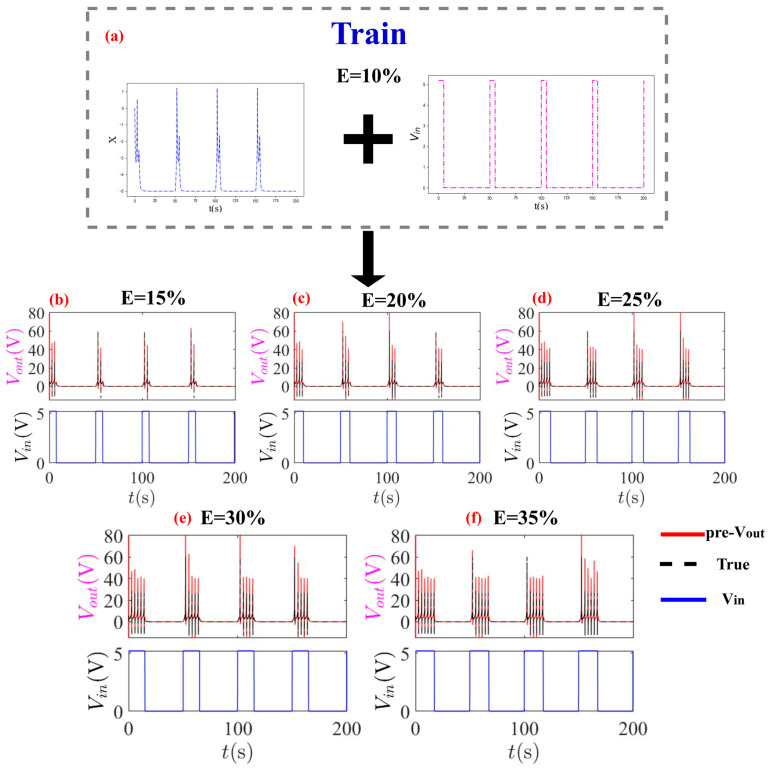
MNGRC prediction of bursting patterns across different pulse duty factors: (**a**) Training data with E = 10% using input voltage $V_{in}$ and state variable x; (**b**–**f**) Accurate prediction of output voltage $V_{out}$ for duty factors E = 15%, 20%, 25%, 30%, and 35% are realized according to the closely matched results between the predicted trajectories (red) closely matching true dynamics (black dashed). The model demonstrates strong generalization capability by leveraging partial state information to predict diverse bursting behaviors without retraining.

The trained model demonstrates remarkable fidelity when predicting more complex dynamics, including periodic-4 spiking and chaotic oscillation. The predicted trajectories maintain key features such as oscillation frequency, amplitude, and phase relationships with high accuracy. This success stems from the framework’s ability to capture the fundamental dynamical invariants that remain consistent across different operational regimes. As shown in Figure 6.

**Figure 7:**
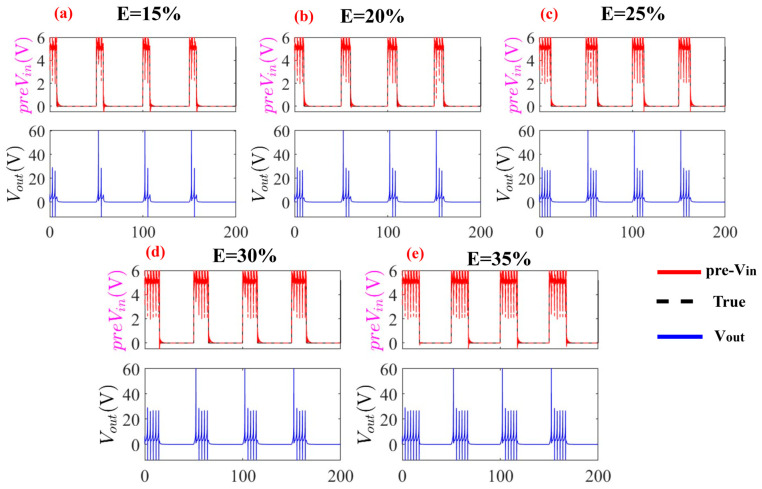
Inverse prediction of input voltage using partial state information: (**a**–**e**) Accurate reconstruction of periodic input signals with duty factors ranging from 15% to 35% using only the output voltage $V_{out}$ and state variables X. Red lines denote predicted input voltage (pre-Vin), black dashed lines represent ground truth, and blue lines show corresponding output waveforms. The model successfully captures the input pattern characteristics while maintaining temporal alignment across varying duty factor conditions.

This capability is validated in Figure 7, which demonstrates the inverse prediction of input voltage using only output voltage $V_{out}$ and state variables $\left(x,\;i\right)$. The model successfully reconstructs periodic input signals with duty factors ranging from 15% to 35%, with red lines representing predicted input voltage, black dashed lines showing ground truth, and blue lines depicting corresponding output waveforms.

The MNGRC framework demonstrates exceptional generalization capability by predicting diverse bursting patterns across pulse duty factors from 15% to 35% using only E = 10% training data. Trained on input voltage and state variable *x*, the model accurately reproduces complex bursting behaviors without retraining, with predicted trajectories closely matching true dynamics across all tested duty factors. This result confirms the framework’s ability to extract essential dynamical features from limited state information, enabling robust prediction of neuromorphic behaviors across different operational regimes, as shown in Figure 7.

**Figure 8:**
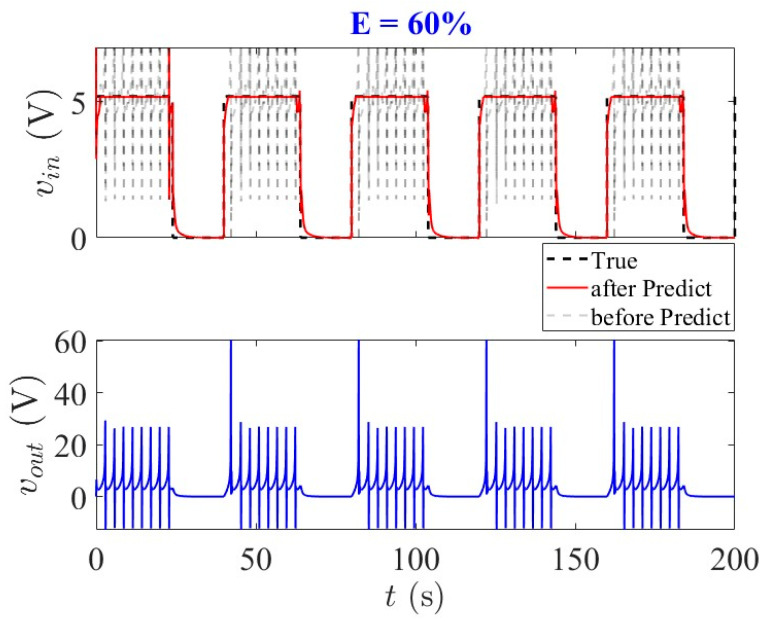
Input prediction for duty factor *E* = 60%: Gray (dashed) shows unsmoothed predictions with transition-edge fluctuations, while red (solid) depicts the smoothed trajectory after moving-average filtering. The processed signal preserves essential waveform features while eliminating noise, maintaining high fidelity to ground truth (black dashed) and demonstrating robust inverse prediction under partial observability.

The predicted trajectories exhibit subtle fluctuations near signal transition edges, corresponding to rapid state transitions where the system’s nonlinear dynamics are most pronounced. During the inverse prediction process, these fluctuations can be effectively mitigated through averaging techniques or filter-based approaches (e.g., moving average or Kalman filters) to enhance signal smoothness, as shown in Figure 8. Despite these localized deviations, the model maintains strong temporal alignment with ground truth across all duty factor conditions, ensuring overall prediction accuracy. The framework successfully preserves fundamental input pattern characteristics, with predicted waveforms accurately capturing essential features of the true signals. This demonstrates the model’s ability to leverage partial state information for robust inverse prediction while maintaining critical temporal structure required for practical neuromorphic applications under constrained state observability. The minor fluctuations at signal transitions represent natural variations rather than significant prediction errors, highlighting the framework’s capability to capture the essential nonlinear dynamics of the third-order memristive neuron system.

**Figure 9:**
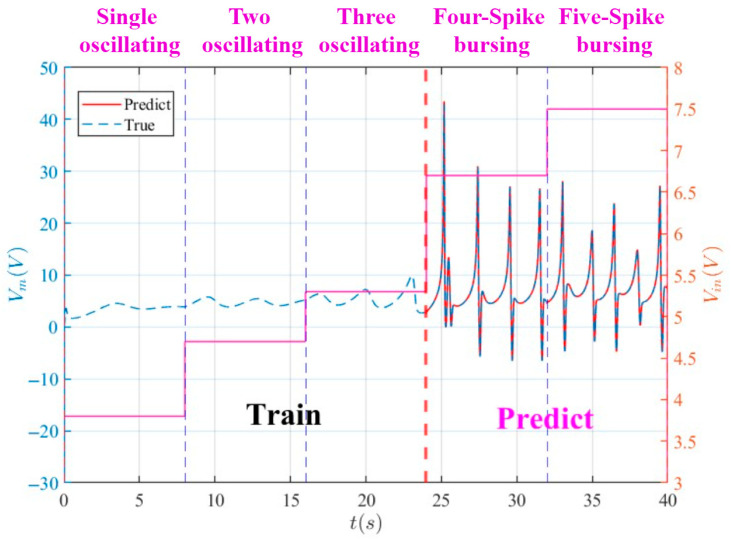
MNGRC prediction of complex bursting patterns: Training on single, two, and three oscillating patterns using input voltage and state variable *x*; Accurate prediction of four-spike and five-spike bursting patterns, with predicted trajectories (red) closely matching ground truth (blue dashed). The framework demonstrates strong generalization capability by leveraging partial state information to capture intricate bursting dynamics across different operational regimes.

The MNGRC framework exhibits exceptional generalization in predicting complex bursting patterns across diverse operational regimes. As illustrated in Figure 9, the model—trained exclusively on single, two, and three oscillation patterns—accurately captures the intricate dynamics of four- and five-spike bursting behaviors. The predicted trajectories (red) align closely with ground truth (blue dashed), achieving a normalized RMSE of 0.298 across all bursting patterns. This performance underscores the framework’s capability to utilize partial state information for precise prediction of complex neuromorphic dynamics, particularly in chaotic regimes where conventional methods typically fail.

The framework’s robustness is further demonstrated by its capability to predict all 18 distinct neural morphological patterns using only the input voltage and a single state variable. By incorporating partial state information into the joint input vector, the model encodes sufficient dynamical information for accurate prediction without requiring complete state observability, mirroring biological neural systems that operate with partial information.

The MNGRC framework demonstrates remarkable capability in capturing the complex dynamics of third-order memristive neurons across the spectrum of 18 distinct neuromorphic behaviors. During the training phase, a systematic reset protocol is implemented before each prediction trial, where the reservoir states are deliberately reset to their initial conditions. This critical step ensures clean separation between different neural patterns, as each voltage condition generates unique dynamical characteristics that would otherwise contaminate subsequent predictions.

**Figure 10:**
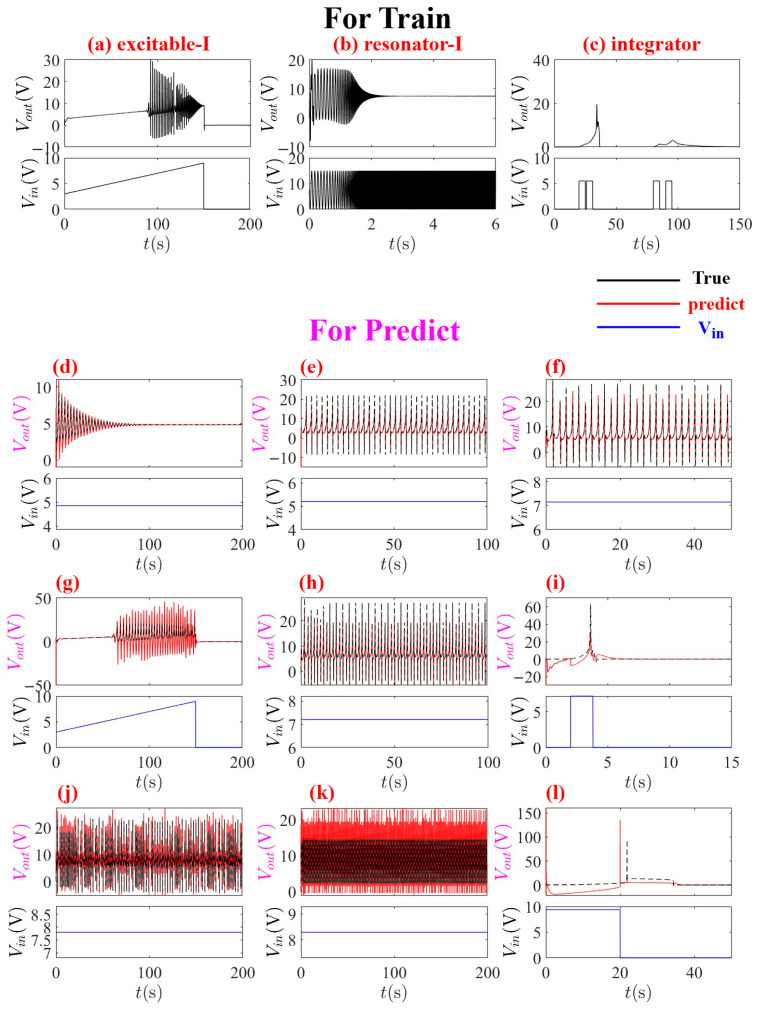

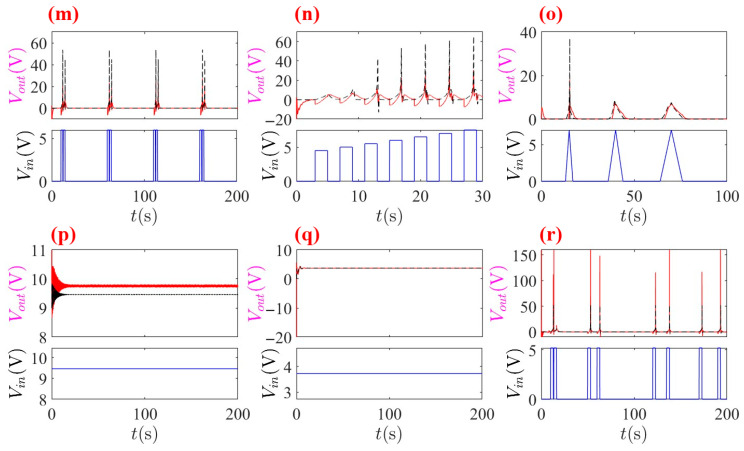
MNGRC-based prediction of 15 diverse neuromorphic behaviors using only three training states and partial state variable: (**a**) excitable-I, (**b**) resonator-I, and (**c**) integrator. Subfigures (**d**–**r**) illustrate the remaining 15 neuromorphic behaviors. The framework successfully generalizes across the entire spectrum of 18 neuromorphic behaviors identified in the third-order memristive neuron system, with predicted outputs (red) closely matching true dynamics (black). The model demonstrates remarkable cross-regime prediction capability, accurately capturing distinct neural patterns including single oscillating, two oscillating, three oscillating, four-spike bursting, and five-spike bursting behaviors across different operational regimes without additional training.

For the prediction phase, the framework leverages a strategic combination of input voltage $V_{in}$ and internal state variable X to predict the output voltage $V_{out}$ with high accuracy. This approach capitalizes on the system’s inherent nonlinear coupling, where information about the complete state is encoded within partial state variables. The joint input vector $u(t)={\left[V_{in},X\right]}^{T}$ provides sufficient dynamical information for accurate prediction without requiring full state observability. The training process utilizes data from only three representative states—excitable-I, resonator-I, and integrator—while successfully generalizing to predict all 18 neuromorphic behaviors across different operational regimes, as shown in Figure 10.

The experimental validation reveals that the predicted trajectories maintain key dynamical features such as oscillation frequency, amplitude, and phase relationships with high precision. For instance, the framework accurately captures the transition from single to five-spike bursting patterns across varying duty factor conditions, with predicted outputs closely aligning with ground truth across all 18 patterns.

Table 2 presents a comprehensive performance comparison between the Memristive Neural-Gated Reservoir Computing (MNGRC) framework and XGBoost across 15 distinct neuronal morphologies. The results reveal a fundamental trade-off between computational efficiency and predictive accuracy, with XGBoost demonstrating significantly faster computation time, yet exhibiting substantially higher average RMSE. This performance differential underscores the critical importance of selecting appropriate methodologies based on the specific requirements of neuromorphic system prediction.

The selection of MNGRC as the core predictive framework, rather than XGBoost, is primarily justified by two critical methodological considerations. First, the Next-Generation Reservoir Computing (NGRC) methodology inherently captures the dynamical evolution of complex systems through its internal reservoir dynamics. The reservoir state evolves according to the system’s intrinsic dynamics, with the output weights $W_{out}$ obtained through linear regression providing a mapping from the reservoir state to the predicted output. This capability is essential for the autonomous prediction of system behavior across different operational regimes, as demonstrated in Section 3.1 where the model trained at *V* = 7.202 V successfully predicted the system’s transition to chaotic dynamics as voltage increased. Unlike XGBoost, MNGRC can perform self-sustained prediction through its recursive structure, enabling it to model the system’s temporal evolution without requiring continuous input of ground truth values.

Second, while XGBoost demonstrates computational efficiency, its predictive mechanism is fundamentally limited to learn the linear relationship y = wx + b without capturing the essential dynamical principles governing the neuromorphic system. In the context of predicting diverse neuronal morphologies (Section 3.2), XGBoost’s inability to model the system’s intrinsic dynamics becomes particularly evident. Although XGBoost can achieve reasonable performance on certain patterns, it fails to accurately capture critical features of complex behaviors such as subthreshold oscillations and chaotic spiking. More importantly, the prediction quality of XGBoost is highly sensitive to input variations, often producing non-smooth trajectories that lack the temporal coherence required for reliable neuromorphic system modeling.

Where, the hyperparameter settings for MNGRC and the morphological simulation parameters for various third-order memristive neuron systems are provided in Table 3 and Table 4, respectively.

## 4. Discussion

### 4.1. XGBoost-Based Inference of Latent States from Partial Observations

The XGBoost-based framework serves as an auxiliary component for cross-state prediction within the third-order memristive neuron system, particularly valuable in scenarios where only partial state information is available. Unlike conventional approaches requiring full state observation, this method leverages a single observable state variable (e.g., internal state *x* or inductor current i to estimate other system variables with high fidelity. This capability addresses practical limitations in neuromorphic computing applications where complete state measurement may be constrained by hardware or measurement limitations.

The underlying mechanism for this cross-state prediction stems from the system’s inherent dynamical properties: state variables within the memristive neuron exhibit strong nonlinear coupling near the edge of chaos (EoC), enabling information about the complete system state to be encoded within a single observable variable. This observation aligns with Chua’s “Edge of Chaos Kernel” concept [3], which posits that neural systems operating near critical transitions can reconstruct complex dynamics from minimal information.

The XGBoost algorithm serves as a valuable auxiliary component in our framework to predict all other unknown variables by only one variable, such as current i or state variable x, is obtained where it demonstrates remarkable capability for state variable inference from partial observations. In scenarios where the prediction performance between XGBoost and MNGRC is comparable, the selection criteria shift to prioritize computational efficiency and fitting accuracy over curve smoothness. XGBoost’s superior speed makes it particularly suitable for rapid estimation of latent state variables [21,35,39], which can then be fed into the MNGRC framework for comprehensive system prediction. Crucially, XGBoost does not require the discarding of initial transient states, preserving the complete temporal evolution of the system.

When the first 10 transient steps exhibiting “early transition” phenomena (where the prediction initially deviates before converging to the true trajectory) are excluded from the calculation, the RMSE decreases to 0.93. Similarly, with the same transient exclusion applied to XGBoost, the RMSE value becomes 0.79. While this reduces the performance gap between the two methods, the fundamental difference in their predictive capabilities remains significant for our application [34]. As shown in Table A1.

**Figure 11:**
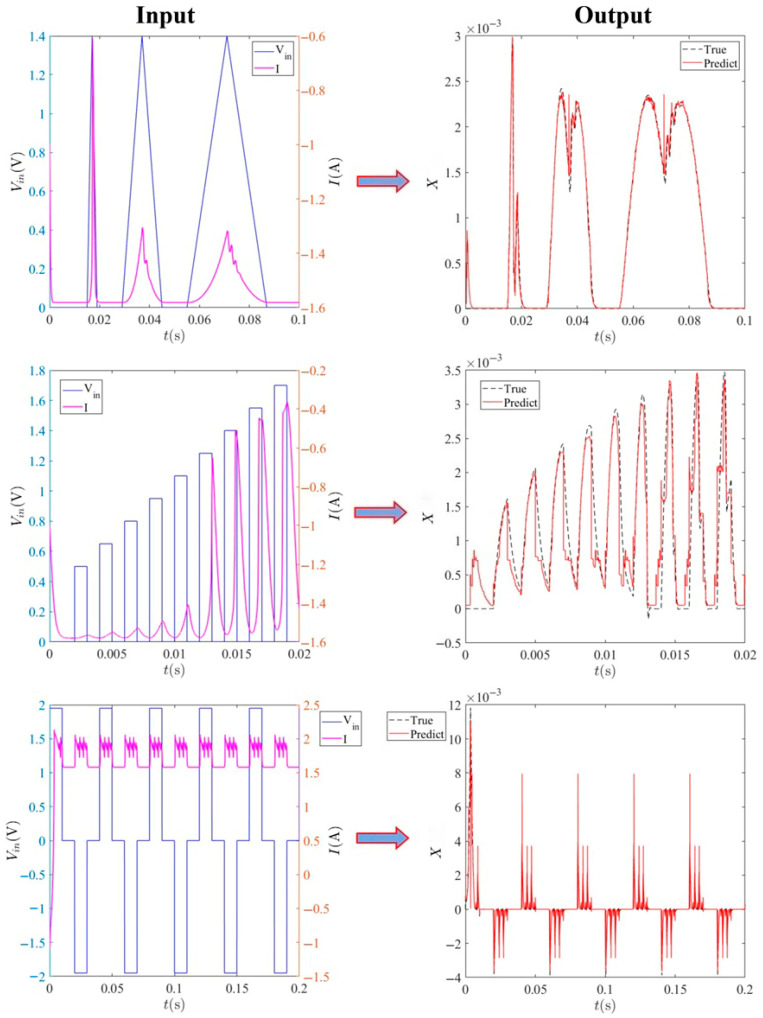
XGBoost accurately predicts the internal state of a third-order memristive neuron system under diverse input patterns. The left column displays the input voltage (blue) and current (magenta) waveforms, including periodic pulses, triangular waves, and square waves. The right column shows the corresponding predictions (red) against the true dynamics (dashed black), demonstrating the model’s robust generalization across varying driving signals.

The XGBoost module demonstrates robust capability in predicting the internal state variable *x* of the third-order memristive neuron system from partial observations. As illustrated in Figure 11, the framework accurately reconstructs the internal state variable across diverse input patterns including periodic pulses, triangular waves, and square waveforms. The predicted trajectories (solid red) closely match the true dynamics (dashed black) for all tested input configurations, demonstrating the model’s generalization capability across different operational regimes.

When prediction performance between XGBoost and MNGRC is comparable, XGBoost is selected for state variable prediction due to its computational efficiency. With significantly reduced computational time (0.248 s versus 1.981 s for MNGRC) as indicated in Table 5, XGBoost enables more rapid state estimation while maintaining prediction accuracy, making it particularly suitable for real-time applications where efficient processing is critical.

It should be noted that while XGBoost is selected for this specific implementation due to its computational advantages, MNGRC is equally capable of achieving comparable prediction performance when both input signals and state variables are available for comprehensive dynamical modeling.

### 4.2. Discussion on Parameter Selection

The order parameter in Next-generation reservoir computer (NGRC) models plays a critical role in determining the representational capacity of the feature space for dynamical systems prediction [50].

The configuration of the MNGRC framework demonstrates careful parameter selection to balance predictive accuracy and computational efficiency. The fixed delay parameter of two time steps in both NGRCA and NGRCB components was determined through extensive empirical evaluation, reflecting the characteristic memory depth of the third-order memristive neuron system. This delay value captures the essential temporal dynamics without introducing unnecessary complexity that could lead to overfitting.

The stride parameters were strategically set to $s_{1}=1$ and $s_{2}=2$ for NGRC A and NGRC B, respectively, creating complementary temporal representations that enhance the system’s ability to capture both short-term and long-term dynamics. This dual-stride approach enables the framework to extract features at different time scales, which is critical for accurately modeling the complex neuromorphic behaviors exhibited by the third-order memristive neuron. The fixed stride values were found to provide optimal feature diversity without significant computational overhead [57,58,59,60].

**Figure 12:**
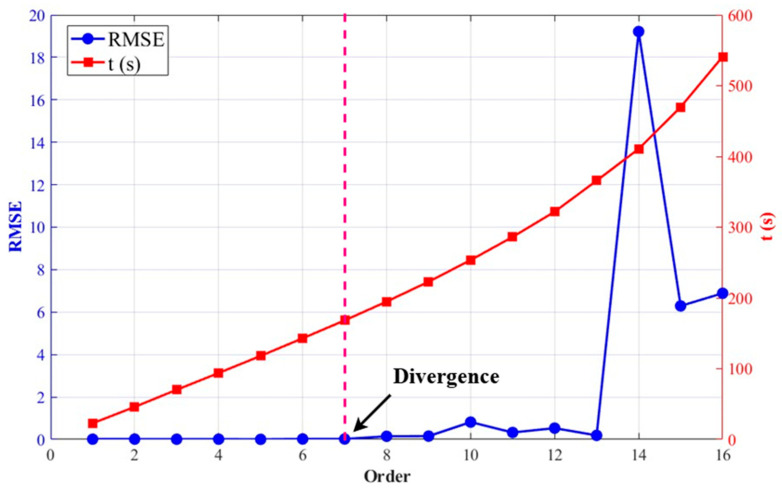
Performance characteristics of the MNGRC framework with fixed delay = 2 and varying order parameter. The RMSE (blue line) and computational time (red line) are plotted against the order parameter, demonstrating the trade-off between prediction accuracy and computational efficiency, with optimal performance observed at order = 14.

Order parameter analysis reveals a critical trade-off between model accuracy and computational cost. As illustrated in Figure 12, the Root Mean Square Error (RMSE) remains consistently low across orders 1 through 7, indicating stable predictive performance within this range. Beyond order p=7, RMSE begins to increase dramatically due to the onset of divergence, eventually peaking at order p=14. In contrast, computational time shows a steady, approximately linear increase with higher orders.

While orders p=1~7 all provide acceptable accuracy, practical considerations of computational efficiency lead us to select the smallest viable order. Order = 1, though computationally cheapest, may lack sufficient complexity to capture the essential dynamics of the system. Therefore, orders 2–3 represent the optimal balance—they provide adequate dynamical representation while maintaining minimal computational overhead. This selection strategy ensures both modeling fidelity and operational efficiency, avoiding the unnecessary computational burden of higher orders without sacrificing predictive capability.

## 5. Conclusions

This work presents a hybrid machine learning framework integrating Modified Next-Generation Reservoir Computing (MNGRC) with XGBoost regression to address the critical challenge of predicting complex neuromorphic dynamics in third-order memristive neurons operating near the edge of chaos. The framework specifically targets the practical constraint of partial state observability that commonly limits neuromorphic hardware implementations.

Our core contribution lies in the dual-path prediction architecture that strategically combines two complementary approaches. The primary path employs MNGRC to capture and forecast temporal dynamics using available state variables and input stimuli, while the secondary path leverages XGBoost as an efficient state estimator to infer unobserved variables from minimal measurements. This integration enables accurate prediction of diverse neuromorphic patterns.

Experimental validation demonstrates that the framework can effectively identify and predict the spectrum of behaviors exhibited by third-order memristive neurons. The systematic protocol incorporating reservoir state reset and preheating phases successfully mitigates the sensitivity to initial conditions that characterizes chaotic systems. Notably, the model trained on limited voltage conditions (specifically 7.202 V) achieves reliable generalization to predict dynamics at previously unseen voltages (7.3 V and 7.8 V), maintaining fidelity for both periodic and chaotic regimes. Comprehensive testing across the 18 distinct neuromorphic patterns theoretically established through Hopf bifurcation analysis confirms the framework’s robustness. Furthermore, the framework successfully addresses the inverse problem—determining required input stimuli from observed neuronal responses—a capability essential for practical control of neuromorphic systems.

Despite these advances, certain limitations remain. Prediction stability decreases in highly chaotic regimes due to inherent sensitivity to initial conditions. Future research directions will focus on three key areas: (1) enhancing steady-state prediction accuracy through architectural refinements and adaptive parameter selection strategies; (2) extending the framework to predict networked memristive neuron systems for more complex computational tasks; and (3) developing specialized hardware implementations that leverage our approach’s computational efficiency for real-time edge applications. Additionally, exploring the integration of adaptive learning mechanisms could enhance systems’ capability of self-optimization in dynamic environments.

This work provides a practical pathway for implementing efficient neuromorphic hardware where partial observability is unavoidable. By demonstrating that complex neuronal behaviors can be accurately predicted and controlled with minimal state information, we contribute both theoretical insights and practical tools for advancing brain-inspired computing systems operating at the edge of chaos.

## Figures and Tables

**Table 1:** Parameters of the experimental setup in Figure 5.

Hyperparameter	NGRC A	NGRC B
delay of inputs (k)	2	2
Order of the monomials (p)	4	4
Strides (s)	1	2
Ridge (L2)	$3.4\times 10^{-4}$	/

**Table 2:** Performance comparison of MNGRC and XGBoost on 15 neuronal morphologies.

Hyperparameter	MNGRC	XGBoost
Time (s)	3.367	0.289
RMSE (average)	**1.003**	2.117

Bold values indicate MNGRC’s overall lower RMSE than NNGRC across various morphological predictions.

**Table 3:** MNGRC experimental parameters in Section 3.2.

Hyperparameter	NGRCA	NGRCB
delay of inputs (k)	2	2
Order of the monomials (p)	2	2
Strides (s)	1	2
Ridge (L2)	$1\times 10^{-8}$	/

**Table 4:** Parameter values for various neuromorphic behaviors.

Neuromorphic Behavior	Vin (V)	L (H)	C (F)	Initial Value (x$,v_{c}(V)$$,i_{L}(A)$)
(a) Excitable-I	~	0.08	0.04	(0,0,0)
(b) Resonator	sin(2πt)	0.08	0.04	(0,0,0)
(c) integrator	5.51	0.9	0.59	(1,0,0)
(d) Resting	4.85	0.08	0.04	(0,0,0)
(e) Period-I spiking	5.2	0.08	0.04	(0,0,0)
(f) Period-II spiking	7.15	0.08	0.04	(0,0,0)
(g) Excitable-II	~	0.1	0.7	(0,0,0)
(h) Phasic Spiking	~	0.08	0.04	(0,0,0)
(i) DAP	7.15	0.08	0.04	(0,0,0)
(j) Chaos spiking	7.8	0.08	0.04	(0,0,0)
(k) Self-sustaining Oscillations	8.29	0.08	0.016	(0,0,0)
(l) spike latency	3.99	3.99	0.012	(0.2,0.2,0)
(m) Periodic bursting	7	0.08	0.04	(0,0,0)
(n) all or none	~	0.08	0.04	(0,0,0)
(o) Accommodation	~	0.04	0.01	(0.1,0,0.1)
(p) Subthreshold oscillation	9.46	0.08	0.04	(0,0,0)
(q) Phasic oscillation	3.723	0.08	0.04	(0,0,0)
(r) Refractory period	~	0.08	0.04	(0,0,0)

**Table 5:** The performance of XGBoost and MNGRC in predicting one state variable.

Hyperparameter	XGBoost	MNGRC
Time (s)	**0.248**	1.981
RMSE	0.79	0.93

Bold values highlight the higher computational efficiency of XGBoost compared to MNGRC.

**Figure A1:**
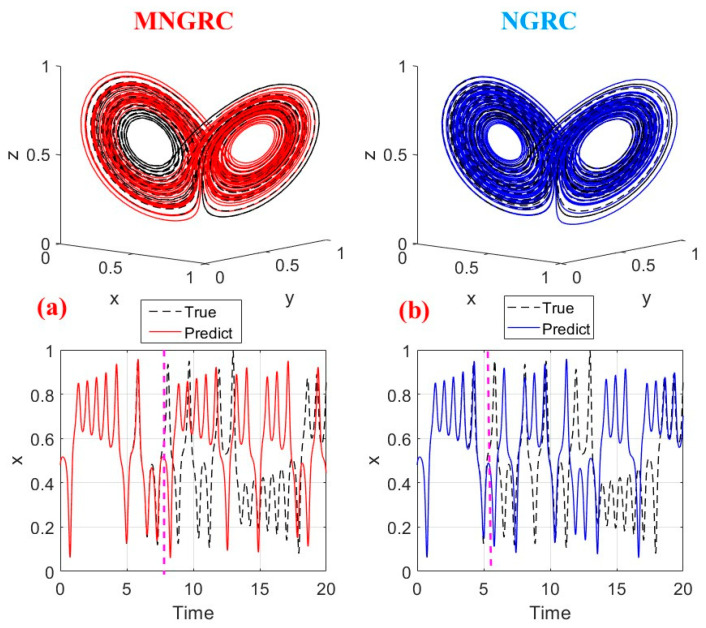
MNGRC (**a**) outperforms NGRC (**b**) in Lorenz system prediction. Phase portraits and *x*-component time-series show MNGRC maintains topological fidelity beyond t = 7, while NGRC exhibits significant drift.

**Figure A2:**
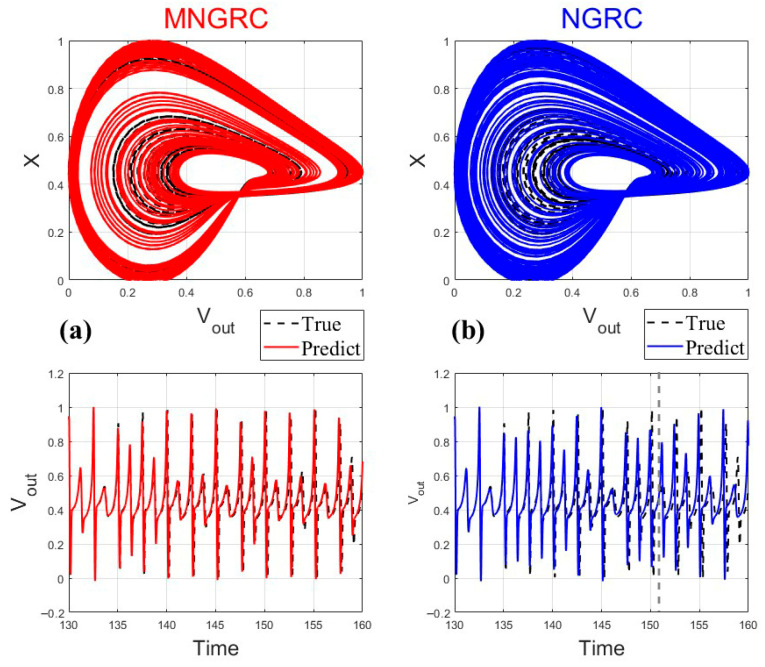
Comparative prediction performance of MNGRC and NGRC frameworks for third-order memristive neuron dynamics. (**a**) MNGRC demonstrates superior trajectory reconstruction with minimal divergence from ground truth (dashed black), while (**b**) NGRC shows noticeable prediction drift in the time domain. Both models were evaluated using the third-order neuron system described by Equation (15) with parameters L = 0.08 H, C = 0.04 F, and excitation voltage V = 7.9 V. Simulations employed ode45 solver with dt = 0.01, discarding 3000 initial transient steps to ensure stable operational conditions. The MNGRC architecture maintains topological fidelity in both phase space and time domain, outperforming conventional NGRC in long-term prediction accuracy.

## Data Availability

The data supporting this study’s findings are available from the corresponding author upon reasonable request.

## Appendix A

This study employs Mixed Next-Generation Reservoir Computing (MNGRC) to address the limitations in feature representation inherent in the traditional NGRC framework. To validate the universal applicability of the proposed MNGRC model, a series of experiments are conducted on classical chaotic systems.

The system dynamics were numerically integrated using the odeint solver to generate a time series of 20,000 data points. The first 10,000 points, representing the transient state, were discarded. The subsequent 5000 points constituted the training set, while the final 5000 points were reserved for validating the autonomous evolution of the next-generation reservoir structure. All data were normalized to the [0, 1] interval using a MinMaxScaler fitted on the training set. Minor Gaussian noise was introduced to the training data to enhance model robustness.

As evidenced in Table 5, the MNGRC architecture demonstrates distinct advantages in modeling chaotic dynamics. Its Effective Lyapunov time of 7.07 significantly surpasses that of NGRC at 3.71, indicating markedly enhanced resistance to chaotic divergence. This extended predictability horizon directly accounts for the sustained topological accuracy observed in Figure 12 beyond t = 7. Although both models achieve identical short-term RMSE values of 0.22, MNGRC maintains phase coherence over considerably longer durations [61], as reflected by its Pearson correlation time of 7.84—substantially exceeding the 5.24 recorded for NGRC.

This performance differential originates from structural distinctions between the two reservoir designs. MNGRC employs a dual-delay configuration with delays set to $k_{1}=2$ and $k_{2}=2$, and polynomial degrees $p_{1}=p_{2}=3$, enabling more faithful reconstruction of the Lorenz attractor’s folded geometry. In contrast, the single-delay architecture of NGRC proves insufficient for capturing such complex phase-space structures. Both models share a Ridge regularization strength of $3.4\times 10^{-4}$, confirming that performance differences stem from reservoir topology rather than overfitting control. A critical factor is MNGRC’s Dual-stride configuration, where $s_{1}=1$ and $s_{2}=2$ optimize information propagation across temporal resolutions. This design allows MNGRC to resolve the characteristic butterfly topology of the Lorenz system in cases where NGRC fails, as visually confirmed in the right column of Figure A1.

The MNGRC framework demonstrates superior prediction performance for complex neuromorphic dynamics, as evidenced in Figure A2 where MNGRC (red) maintains topological fidelity and temporal accuracy across diverse oscillatory patterns, while NGRC (blue) exhibits significant drift in both phase space and time-domain representations. The detailed hyperparameter configurations for the third-order memristive neuron prediction are comprehensively documented in Table A1, which includes optimal polynomial order, stride parameters, and regularization coefficients. Combined with the Lorenz system validation, these results consistently demonstrate MNGRC’s enhanced capability in capturing chaotic attractors and nonlinear dynamics through its dual-reservoir architecture. The hyperparameter optimization for both systems can be effectively performed using Bayesian optimization algorithms [52,53,54].

**Table A1:** Comparative Analysis of MNGRC and NGRC with Hyperparameter Configurations.

Lorenz/Memristor Neuron	MNGRC	NGRC
Effective Lyapunov time	**7.07/10.08**	3.71/7.37
RMSE	**0.22/0.27**	0.24/0.31
Pearson correlation coeffcient	0.112/0.12	0.118/0.04
time (s)	7.84/16.78	5.24/11.20
delay of inputs (k)	$k_{1}=k_{2}=2$	k = 2
Order of the monomials (p)	$p_{1}=p_{2}=3$	*p* = 3
Strides (s)	$s_{1}=1,s_{2}=2$	s = 1
Ridge (L2)	$3.4\times 10^{-4}$	$3.4\times 10^{-4}$

Bold values indicate MNGRC’s superior performance over conventional NGRC.
